# Real-Time and In-Flow Sensing Using a High Sensitivity Porous Silicon Microcavity-Based Sensor

**DOI:** 10.3390/s17122813

**Published:** 2017-12-05

**Authors:** Raffaele Caroselli, David Martín Sánchez, Salvador Ponce Alcántara, Francisco Prats Quilez, Luis Torrijos Morán, Jaime García-Rupérez

**Affiliations:** Nanophotonics Technology Center (NTC), Universitat Politècnica de València, 46022 Valencia, Spain; rcaroselli@ntc.upv.es (R.C.); damarsa5@ntc.upv.es (D.M.S.); salponce@ntc.upv.es (S.P.A.); frapraqu@ntc.upv.es (F.P.Q.); luitorm2@ntc.upv.es (L.T.M.)

**Keywords:** photonic sensor, porous silicon, Bragg reflector, microcavity, real-time sensing, in-flow sensing

## Abstract

Porous silicon seems to be an appropriate material platform for the development of high-sensitivity and low-cost optical sensors, as their porous nature increases the interaction with the target substances, and their fabrication process is very simple and inexpensive. In this paper, we present the experimental development of a porous silicon microcavity sensor and its use for real-time in-flow sensing application. A high-sensitivity configuration was designed and then fabricated, by electrochemically etching a silicon wafer. Refractive index sensing experiments were realized by flowing several dilutions with decreasing refractive indices, and measuring the spectral shift in real-time. The porous silicon microcavity sensor showed a very linear response over a wide refractive index range, with a sensitivity around 1000 nm/refractive index unit (RIU), which allowed us to directly detect refractive index variations in the 10^−7^ RIU range.

## 1. Introduction

Nowadays, analysis applications require the use of sensing devices with a higher sensitivity, suitable to realize a detection with an as-short-as-possible response time [1,2]. In this context, porous silicon (PS) sensing structures have been playing an increasingly important role in these fields during the last decade. On the one hand, PS can provide very high sensitivity due to the fact that the sensing interaction takes place directly inside the photonic structure itself [3]. Additionally, its high surface-to-volume ratio improves surface functionalization, as it detects a higher amount of biomolecular interactions [4]. These advantages are due to the particular morphology of PS, composed of a network of pores entering into the silicon that provide a higher interaction volume and an enormous internal surface [5]. Moreover, PS can be formed simply, quickly and inexpensively since it is the result of the electrochemical etching of a silicon substrate.

PS has been demonstrated to be a very versatile substrate, making it suitable for the creation of many different configurations of sensing devices, including electrical and optical sensors [6]. With regards to optical sensing, PS has been used to realize many different configurations of sensing structures [7,8], including single layers, Fabry Perot (FP) filters [9,10,11], double layers (DLs) [12], multilayers, such as Bragg reflectors (BRs) [13,14], optical microcavities (MCs) [15,16,17,18,19,20,21,22] and surface gratings [23,24]. Some of those configurations have also been implemented in a membrane format by removing the silicon substrate below the PS structure [25,26,27,28]. Recently, it has also been demonstrated that PS can be used as a substrate for the creation of planar photonic structures such as Mach-Zehnder interferometers [29] or ring resonators (RRs) [30,31].

In this work, we present our experimental development of a porous silicon microcavity (PSMC) based sensor for high-sensitivity, real-time and in-flow sensing applications. Section 2 describes the design, the fabrication and the characterization of such structure, as well as the experimental opto-fluidic setup developed to carry out the experiments and to monitor in-continuum the evolution of the photonic structure spectrum. In Section 3, the experimental results are reported and discussed. In this respect, we studied the sensitivity of the PSMC to refractive index (RI) variations of the bulk medium by flowing several ethanol (EtOH) dilutions and monitoring the spectral shift in real-time. A sensitivity around 1000 nm/RIU (refractive index unit) and a minimum detected RI variation in the 10^−7^ RIU range have been achieved.

## 2. Materials and Methods

### 2.1. PSMC Design

Our sensing structure consisted of an optical microcavity created between two Bragg reflectors. A Bragg reflector is a periodic structure formed by alternating layers of high (n_H_) and low (n_L_) RI and provides a reflectivity spectrum characterized by the presence of a photonic band gap (PBG) centered on the Bragg wavelength, λ_B_. The thickness of the layers, d_H_ and d_L_, satisfies the relation 2(n_H_·d_H_ + n_L_·d_L_) = m·λ_B_, where m is the order of the Bragg condition. The microcavity structure can be obtained by placing a λ_B_/2 layer between the two Bragg reflectors, giving rise to the appearance of a resonance peak inside the PBG. This peak will be used to perform the sensing experiments, since its position will depend on the RI of the substance infiltrated into the pores of the PSMC structure.

In order to increase the sensitivity of the PSMC structure, high porosities of the layers are required, as this will increase the total volume where the target substance is infiltrated. Additionally, for the case of biosensing purposes, a combination of a high porosity and a low pore diameter allows a higher surface-to-volume ratio, which also allows the immobilization of a higher amount of biomolecular receptors, in order to detect more biosensing events. On the other hand, having an extremely high porosity of the layers will make the structure more fragile and easily collapsible. Therefore, we selected a maximum porosity value for the layers of 85% in order to have an as-high-as-possible sensitivity while keeping the structure robust.

A model based on the Transfer Matrix Method (TMM) [32,33] was used to determine the structural parameters of the PSMC. This model allowed us to calculate the transmission and the reflection spectra of a one-dimensional structure, which consisted of an arbitrary number N of alternating porous silicon layers with different porosities and thicknesses. For the calculations, the porosity and the RI of the layers were related using the Bruggeman equation [34].

**Figure 1:**
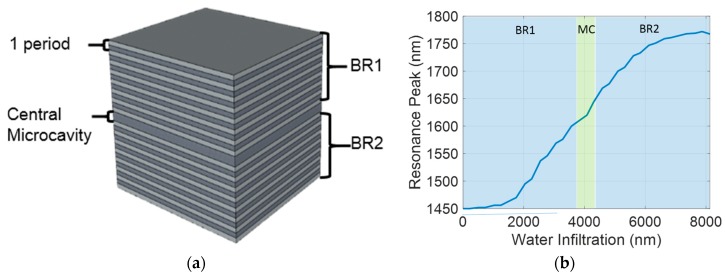
(**a**) Scheme of the porous silicon microcavity (PSMC) used in this work. (**b**) PSMC resonance peak position depending on the water infiltration depth.

We carried out the design of the PSMC considering the maximum porosity previously indicated, as well as the MC created between the two Bragg reflectors (BR1 and BR2), using a high porosity layer (with index n_L_) and having a thickness of 2h_L_. The sequence considered for the PSMC structure, which is schematically depicted in Figure 1a, was [n_H_, n_L_] × 7, n_H_, n_L_ − MC, n_H_, [n_L_, n_H_] × 7. Since our working conditions were λ = 1550 nm and T = 25 °C, for the simulation and experimental stages, we considered the RI value of water to be 1.3173, obtained from [35]. In the simulations, we also took into account the infiltration of the target solution into the pores [36]. Under ideal conditions, the target solution flowing over the sensor should completely fill the pores of the PSMC structure. However, for small pore diameters (as in our case, which will be described in the section corresponding to the fabrication of the PSMC structure), the air initially filling the pores is not able to completely leave the pores, resulting in a partial infiltration of the target substance on the structure. Figure 1b shows an example of the evolution of the resonance peak position of the PSMC, depending on its water infiltration. We can see that the resonance shift is minimum when water infiltrates the substance, until approximately the middle of the BR1. After this point, the position of the resonance peak increases drastically with an almost linear behavior, until approximately the middle of the BR2. Finally, the effect of filling the deeper half of the BR2 has a minimum influence on the displacement of the resonance peak. Therefore, it is worth noting that the infiltrated liquid shifts the resonance peak, even when it has not reached the MC layer itself.

**Figure 2:**
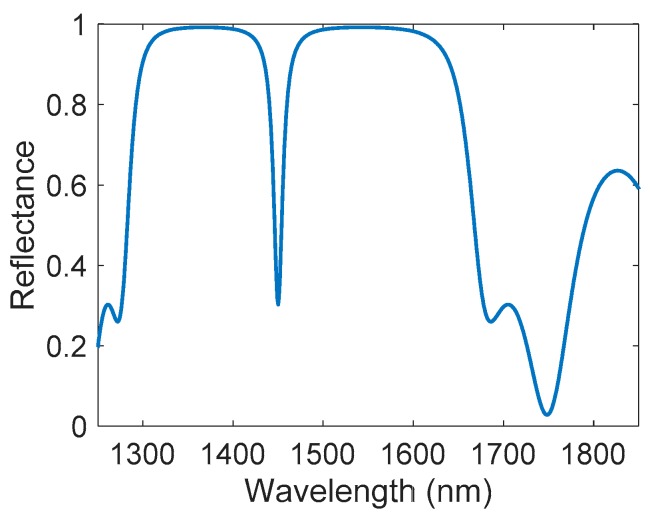
Simulated reflectance spectrum of the designed PSMC, having a cavity peak located at 1450 nm for an air medium.

The RIs, and the corresponding thicknesses of the PSMC layers selected from the calculations carried out, are n_H_ = 1.63, n_L_ = 1.26, d_H_ = 220 nm and d_L_ = 288 nm. These RIs correspond to a porosity of 67% and 83% for the n_H_ and the n_L_ layers, respectively. The calculated reflectance spectrum of the simulated structure presents a peak in 1450 nm for an air medium, as depicted in Figure 2.

### 2.2. PSMC Fabrication

The PSMC was fabricated by an electrochemical etching of a single-side, polished, boron-doped <100> oriented c-Si wafer, with an electrical resistivity of 0.01–0.02 Ω·cm. The solution used to produce the PS was composed using 48% hydrofluoric acid (HF) and 99% EtOH, in a 1:2 relation. In order to remove possible organic residues on the surface, before the etching process, the silicon wafer was cleaned using a piranha solution (volume ratio H_2_SO_4_:H_2_O_2_ = 3:1) at room temperature. Afterwards, the wafer was rinsed with deionized water (DIW). Finally, in order to remove residual surface impurities, it was cleaned with acetone, isopropyl alcohol and DIW. The protocol used to carry out the electrochemical etching process to prepare the PSMC, and the physical characteristics of the n_H_ and n_L_ layers fabricated are presented in Table 1. In preparing each layer of porous structure, the most important parameters taken into account were the HF concentration [HF], the current density J, and the etching time T.

### 2.3. PSMC Physical and Optical Characterization

**Figure 3:**
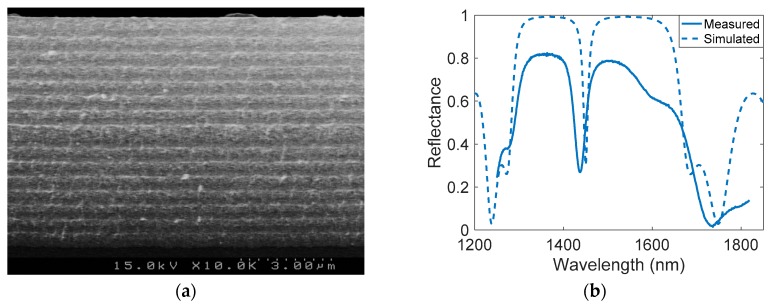
(**a**) SEM cross-sectional image of the fabricated PSMC. (**b**) Comparison between the measured Fourier transform infrared (FTIR) reflectance spectrum of the fabricated PSMC and the simulated reflectance spectrum for the designed PSMC.

**Figure 4:**
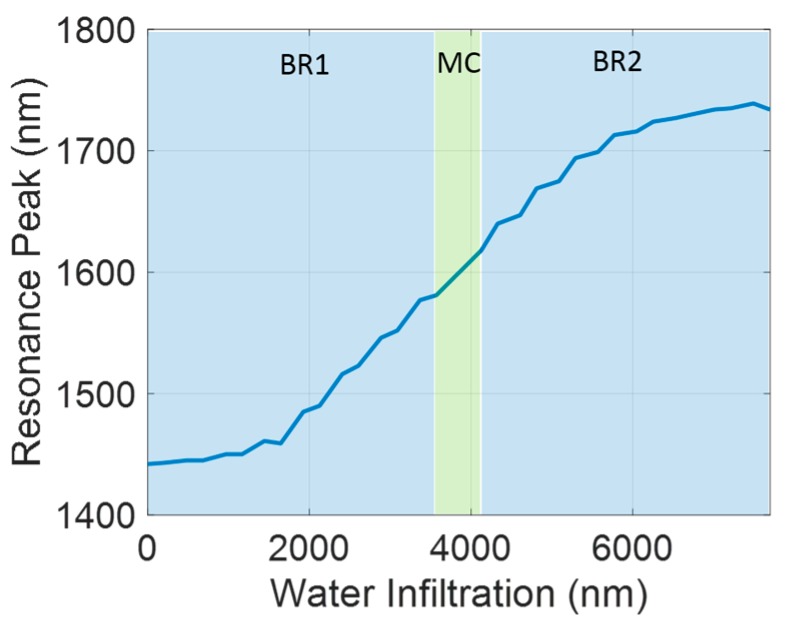
PSMC resonance peak position depending on the water infiltration depth, simulated with experimental porosity values.

Figure 3a shows a scanning electron microscope (SEM) cross-sectional image of the PSMC, where the light and dark grey layers are the n_H_ and the n_L_ layers, respectively. The average measured thicknesses of both layers are 202 and 279 nm, respectively. The overall thickness of the PSMC is around 7700 nm. The ImajeJ software [37] was used in order to obtain the average porous diameter, which is in the range of 10 nm. Figure 3b shows the PSMC reflectance spectrum characterized, using a Fourier transform infrared (FTIR) spectrometer. The experimental RIs of the layers were calculated fitting the reflectance spectra with the TMM-based model. The obtained RIs were n_H_ = 1.7 and n_L_ = 1.39, corresponding to porosities of 65% and 77%, respectively. Such experimental results are in a good agreement with those calculated in the design phase. As observed, the spectrum presents a resonance peak in 1440 nm for an air environment, as expected from the design stage. The evolution of the resonance position, depending on the water infiltration, has also been calculated for the structural parameters of the fabricated sample and is depicted in Figure 4.

**Figure 5:**
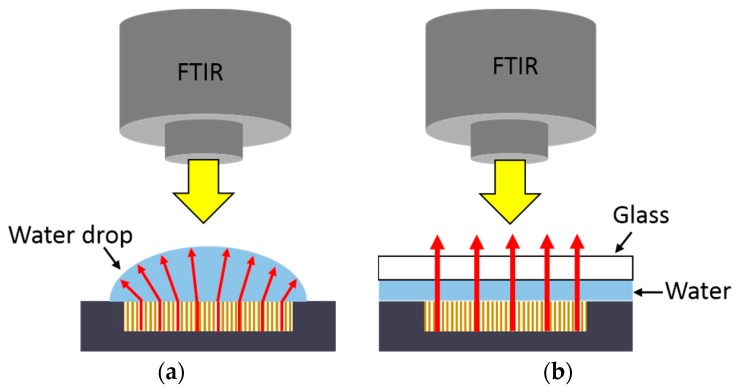
Schematic representation of the direction of the light emitted by the FTIR and reflected by the PSMC sample when (**a**) a deionized water (DIW) drop is deposited on top of the porous structure and (**b**) when a glass sample-carrier is placed on top of the DIW drop.

**Figure 6:**
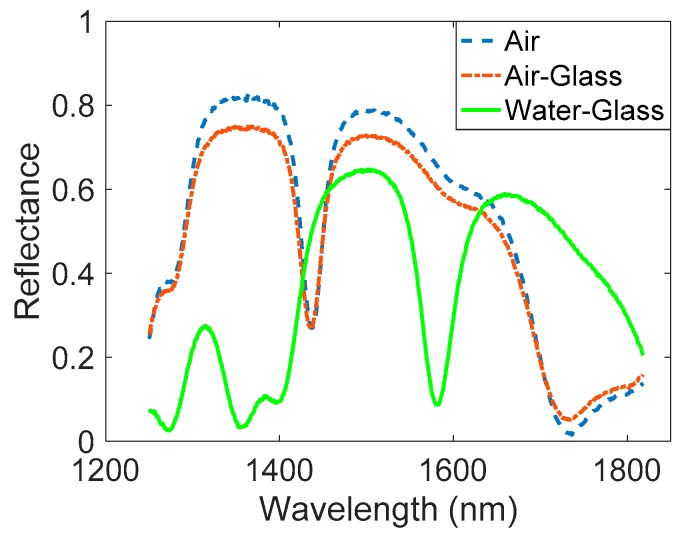
FTIR reflectance spectrum of the PSMC in air, in air with the glass sample-carrier placed on top of the PSMC sample, and with a DIW film between the PSMC sample and the glass.

After the initial characterization in air, a drop of DIW was deposited on the porous sample. As depicted in Figure 5a, the diffuse part of the reflected light took relevance due to the water surface curvature. Because of this, it was not possible to characterize the real specular reflectance with the FTIR. In order to solve this problem, a glass sample-carrier was placed over the drop of DIW in a planar position, allowing us to obtain the PSMC spectrum in the presence of DIW, as shown in Figure 5b. Figure 6 shows the FTIR reflectance spectrum of the PSMC sample in air, with and without the glass sample-carrier placed on top of it, as well as when DIW is placed between the PSMC sample and the glass. Mainly due to its reflectivity, when the glass was placed on top of the sample, the reflectance decreased but the spectrum showed no displacement. When the DIW drop was deposited between the PSMC sample and the glass, the reflectance spectrum shifted. In particular, the resonance peak position shifted to 1580 nm. According to the study of the DIW infiltration degree influence on the resonance peak position depicted in Figure 4, it implies that DIW infiltrated until around the middle of the porous structure due to the impossibility of completely removing the air within the pores, as previously explained.

### 2.4. Experimental Setup

Our objective in this work was to carry out a continuous monitoring of the PSMC sensing response in real-time, which would allow us to detect extremely small RI variations occurring on the sensing structure. Typically, most works focused on the development of PS-based sensors, and would only perform a measurement before and after the target substance was deposited on the sensing structure [22]. To do this, the sensing sample was usually removed from the experimental setup and then re-placed, leading to a significant error in the measurement, and thus limiting the experimental accuracy, especially when very small spectral shifts needed to be measured. In other works, a flow cell was used to flow the substances under analysis, without removing the sample from the experimental setup, in order to remove the source of inaccuracy [28]. This also allowed one to monitor the evolution of the sensing signal at the same time, in order to determine the kinetics of the sensing events being monitored, which also leads to an improvement of the sensing performance. However, the characterization equipment that is typically used provides a very poor spectral resolution, and can require a very long time to acquire each spectrum. This makes it difficult to detect very small spectral shifts with high accuracy, and to obtain a real-time monitoring of the sensing events.

**Figure 7:**
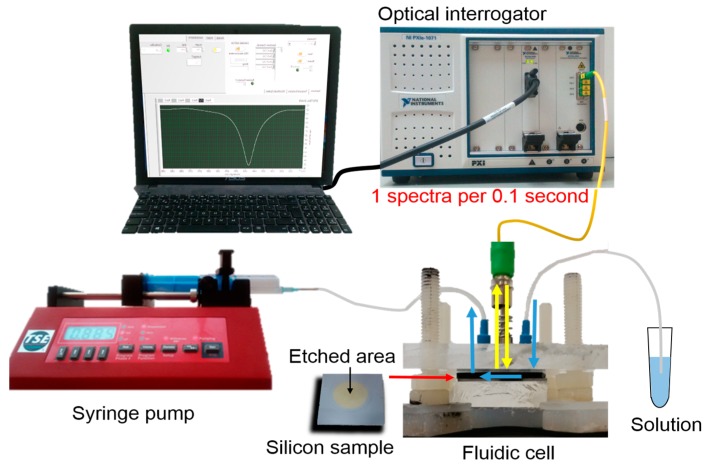
Schematic illustration of the opto-fluidic setup used to carry out the sensing experiments.

In order to overcome these limitations, we developed an opto-fluidic experimental setup, which was able to carry out the sensing measurements in-continuum, in real-time, and with a high spectral resolution. The setup was composed of fluidic and optical parts operating at the same time, in order to flow the reagents, and simultaneously monitor the spectrum evolution. The whole experimental opto-fluidic setup is shown in Figure 7.

**Figure 8:**
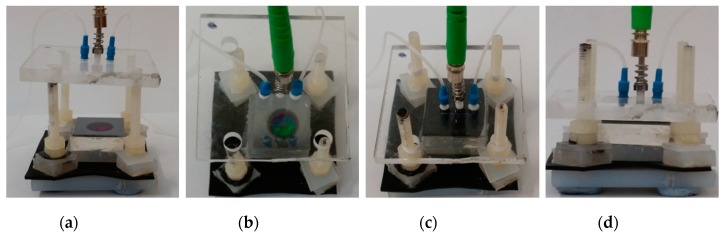
Pictures of the opto-fluidic experimental cell used to flow the solutions over the PSMC sample: (**a**) lateral view open cell; (**b**) top view open cell; (**c**) top view hermetically sealed cell; (**d**) lateral view hermetically sealed cell.

Regarding the fluidic setup, the central element was a homemade fluidic cell, which is shown in Figure 8. Such a cell allowed different solutions to flow over the PSMC sample, while it was simultaneously illuminated with a perpendicularly placed optical fiber. The fluidic cell was basically composed of a 4 × 4 cm polymethyl methacrylate (PMMA) base, a Viton channel and an upper PMMA piece. The PMMA base was the support on which the PS sample was placed. The Viton channel was placed on top of the PS sample, in order to flow the target substance only over the region where the optical interrogation was carried out. The upper PMMA piece was placed on top of the PS-Viton channel system to seal the fluidic channel. In the upper PMMA piece, the input and output fluidic tubes and the optical fiber were attached. Some pressure was applied to the upper PMMA piece to hermetically seal the fluidic system. To reduce the possibility of breaking the PS structure, a polydimethylsiloxane (PDMS) base was placed under the PS sample to absorb the pressure. Furthermore, the PDMS base attenuated any possible external vibrations. When the experiments were carried out, the solutions were flowed using a syringe pump. The pump was configured in withdraw mode in order to generate the vacuum, which sucked the solutions from a vial located at the end of the microfluidic system, thus making the liquid flow through the Viton channel. The withdraw mode allowed us to flow different solutions by simply changing the vial at the end of the system. The pump was set to a constant flow rate of 40 μL/min during the whole experiment.

Regarding the optical setup, the optical fiber was connected to an optical interrogator (OI) PXIe-4844 from National Instruments (Austin, TX, USA). Such an OI is an optical measuring apparatus able to obtain one spectrum every 0.1 s in a wavelength range of 1510–1590 nm with a resolution of4 pm. This apparatus provides the advantage of analyzing the reflected light spectrum in real-time with a very high resolution. A LabVIEW program was implemented to continuously examine the evolution of the reflectance spectrum.

## 3. Results

**Figure 9:**
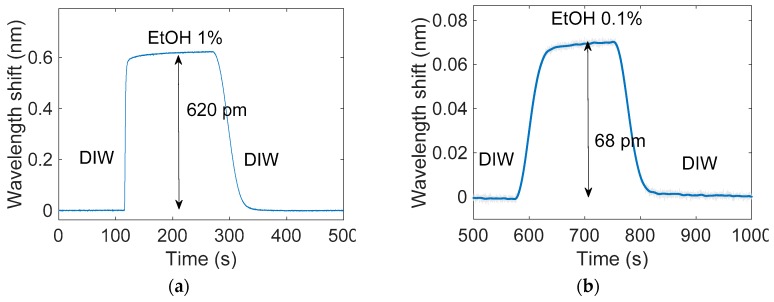

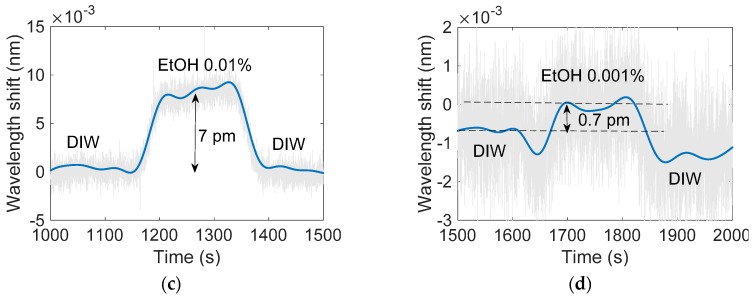
Resonance peak wavelength shift for the EtOH concentrations (**a**) 1%, (**b**) 0.1%, (**c**) 0.01% and (**d**) 0.001%. The raw measured data is shown in light grey, while the evolution after a noise filtering process is shown in blue.

Several RI sensing experiments were performed in order to determine the sensitivity and the detection limit of the PSMC-based sensing system. The experimental sensing procedure consisted of flowing several EtOH dilutions in DIW over the PSMC sensor, and monitoring in real-time the evolution of the PSMC resonance peak when the RI was changed. The EtOH concentrations in DIW in these dilutions were 1%, 0.1%, 0.01% and 0.001%, which means a relative RI change of 6.6 × 10^−4^, 6.6 × 10^−5^, 6.6 × 10^−6^ and 6.6 × 10^−7^ RIU, respectively. Such RI values were obtained by using the model proposed in [35]. The RI sensing experiments were carried out at a constant temperature of 25 °C. The temporal evolution of the resonance peak position for the mentioned EtOH concentrations is shown in Figure 9. The RI changes and the relative shifts for each EtOH concentration are represented in Table 2. A noise in the range of only 0.8 pm was measured in the experiments, which was further reduced by using Discrete Fourier Transform (DFT) algorithm in order to eliminate the high frequency components not related with the sensing, leading to a reduction of the noise by a factor 4x. Because of this process, it was possible to clearly appreciate the spectral shifts that occurred at the lower EtOH concentrations.

**Figure 10:**
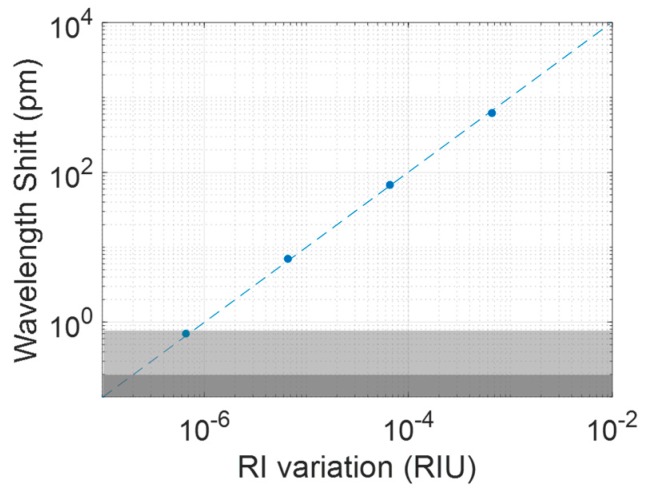
Relationship between the PSMC resonance peak wavelength shift and the RI change in logarithmic scale. The light grey region represents the noise level for the experimental raw data, whereas the dark grey region represents the noise level after the filtering process.

Figure 10 shows the relationship between the PSMC resonance peak wavelength shifts and the RI variation for each EtOH concentration in logarithmic scale as well as the sensitivity curve. As is shown in this graph, the PSMC presents a very linear behavior for the measurement of RI variations over a very wide range of concentrations, allowing to measure values even in the 10^−7^ RIU range, as experimentally demonstrated. The PSMC exhibited a sensitivity around 1000 nm/RIU.

## 4. Discussion

Table 3 summarizes some of the main results reported in the field of the development of PS-based sensing structures, where details about the experimental conditions are also indicated. Regarding the sensitivity to RI variations, our result represents an improvement by a factor of at least ~2x compared to the best results previously reported due to the optimization of the porosity of the structure. Regarding the experimental conditions, only some of the works performed a continuous monitoring of the PS sensing structure, while flowing the target substance over it. All of them made use of a spectrometer to acquire the spectrum, leading to a reported acquisition period of several seconds, a time at least one order of magnitude longer than that reported in this work. Additionally, typical spectral resolutions of those spectrometers are in the range of several hundreds of pm (compared to 4 pm for the optical interrogator used in this work), which also limits the minimum spectral shift that can be accurately measured.

It is also worth noting that some groups have reported experimental sensitivity values above 1000 nm/RIU [25,26]; however, they used a more complex anisotropic configuration of the porous silicon sensing structure, where polarization diversity was required for the interrogation, significantly increasing the complexity of the interrogation and making it difficult to monitor in real-time, as proposed in this work.

## 5. Conclusions

In this work, extremely low RI variations were experimentally detected in real-time by using a porous silicon microcavity as an optical sensor. The experimental results indicate that the optical sensor presents a sensitivity as high as 1000 nm/RIU and a limit of detection in the 10^−7^ RIU range. The achievement of such high sensitivity is determined mainly by the fact that the sensing occurs directly inside the photonic structure. This leads to a higher light–matter interaction, contrary to what occurs for traditional evanescent wave photonic sensing structures, where only a small portion of the optical field interacts with the target substance. Considering this phenomenon, the porosities of the layers were optimized in order to achieve an as-high-as-possible sensitivity, while maintaining the robustness of the structure. Furthermore, such experimental results were obtained in real-time due to the opto-fluidic setup developed to realize the experiments, which allowed us to monitor in-continuum the PSMC spectrum evolution with a very high spectral resolution.

PS’s higher light–matter interaction and greater surface-volume ratio, coupled with the possibility to perform real-time monitoring of the sensing response, make it a suitable platform for the development of high-sensitivity biosensing devices, in which the receptors could be immobilized on the inner surface of the pores.

## Figures and Tables

**Table 1:** Electrochemical etching protocol used to prepare the PSMC.

Layer	[HF]	J (mA/cm^2^)	T (s)
n_H_	16%	16	10.3
n_L_	16%	45	6.9

**Table 2:** Values of the refractive index (RI) changes of the several EtOH concentrations and of the relative PSMC resonance peak wavelength shift.

[EtOH] (%)	ΔRI (RIU)	Δλ (pm)
1	6.6 × 10^−4^	620
0.1	6.6 × 10^−5^	68
0.01	6.6 × 10^−6^	7
0.001	6.6 × 10^−7^	0.7

**Table 3:** Review of experimental results reported using PS-based sensing structures. Several configurations of the PS sensing structures are considered (SL: single layer, FP: Fabry Perot, DL: double layer, BR: Bragg reflector, MC: microcavity, and RR: ring resonator).

PS Structure	Target Substance	Interrogation Equipment	Sensitivity (nm/RIU)	Continuous Flow	Acquisition Period	Reference
MC	Sucrose	Spectrometer	n/a	✓	8 s	[15]
FP	Hydrogen	Spectrometer	n/a	✓	8 s	[11]
MC	Solvents	Spectrometer	425	n/a	n/a	[16]
BR	Gas	Spectrometer	470 ± 40	n/a	n/a	[14]
MC	Solvents	Spectrometer	~550	n/a	n/a	[18]
DL	Proteases	Spectrometer	n/a	✓	2 s	[12]
MC	Ethanol	Spectrometer	~350	✓	4 s	[20]
MC	Solvents	Spectrometer	200	✓	n/a	[21]
RR	NaCl	Tunable laser	380	n/a	n/a	[30]
MC	Streptavidin	Spectrometer	n/a	✓	20 s	[26]
RR	Glucose	Tunable laser	560	n/a	n/a	[31]
MC	Ethanol	Optical interrogator	~1000	✓	0.1 s	This work
